# Helminth infection induces non-functional sensitization to house dust mites

**DOI:** 10.1371/journal.pone.0253887

**Published:** 2021-07-01

**Authors:** Virginie Doyen, Carine Truyens, Hoa Nhu Thi, Hiep Tran Thi Mong, Thanh Le Chi, Frederic De Blay, Phuong Thi Ngoe Huynh, Olivier Michel, Francis Corazza

**Affiliations:** 1 Clinic of Immunoallergology, CHU Brugmann, Brussels, Belgium; 2 Laboratory of Translational Research, ULB223, CHU Brugmann, Université Libre de Bruxelles (ULB), Brussels, Belgium; 3 Parasitology Laboratory, ULB Center for Research in immunology (U-CRI), Université Libre de Bruxelles (ULB), Brussels, Belgium; 4 Parasitology and Mycology Department, Pham Ngoc Thach University of Medicine, Ho Chi Minh, Vietnam; 5 Department of Family Medicine, Pham Ngoc Thach University of Medicine, Ho Chi Minh, Vietnam; 6 Immunology Laboratory, Pasteur Institute, Ho Chi Minh, Vietnam; 7 Chest Diseases Department, Strasbourg University Hospital, Strasbourg, France; 8 Biocluster des Haras, ALYATEC, Strasbourg, France; 9 Department of Hematology, Bordet Institute, ULB, Brussels, Belgium; 10 Laboratory of Translational Research, ULB223, CHU Brugmann, Immunology Laboratory, LHUB-ULB, Université Libre de Bruxelles (ULB), Brussels, Belgium; Auburn University College of Veterinary Medicine, UNITED STATES

## Abstract

**Background:**

IgE characterizes the humoral response of allergic sensitization but less is known about what modulates its function and why some patients present clinical symptoms for a given IgE level and others do not. An IgE response also occurs during helminth diseases, independently of allergic symptoms. This response could be a model of non-functional IgE.

**Objective:**

To study the IgE response against environmental allergens induced during natural helminth infection.

**Methods:**

In 28 non allergic subjects from the periphery of Ho Chi Minh city with (H+, n = 18) and without helminth infection (H-, n = 10), we measured IgE and IgG4 against several components of *Dermatophagoïdes pteronyssinus* (Dpt) and Ascaris (a marker of immunization against nematodes), and determined the IgE component sensitization profile using microarray ISAC biochips. The functional ability of IgE to induce degranulation of cultured mast cells was evaluated in the presence of Dpt.

**Results:**

Non allergic H+ subjects exhibited higher levels of IgE against Dpt compared to H- subjects. Dpt IgE were not functional *in vitro* and did not recognize usual Dpt major allergens. IgE recognized other component allergens that belong to different protein families, and most were glycosylated. Depletion of IgE recognizing carbohydrate cross-reactive determinant (CCD) did not induce a reduction in Dpt IgE. The Dpt IgG4 were not significantly different.

**Conclusion:**

Helminth infections induced IgE against allergens such as Dpt and molecular components that belong to different sources as well as against CCD (such as β-1,2-xylose and/or ⍺-1,3-fucose substituted N-glycans). Dpt IgE were not able to induce degranulation of mast cells and were not explained by sensitization to usual major allergens or N-glycans.

## Introduction

The canonical type 2 (T2) immune response involves immunoglobulin (Ig) E, IgG1, and IgG4 with eosinophils, basophils, mast cells (MCs), and alternatively activated macrophages, as well as interleukin (IL)-4, IL-5, IL-9, and IL-13. In the circulation, IgE is the least abundant class of Ig and is thought to have evolved in mammals from first-line defenses against parasites, which are too large to be phagocytosed. The IgE immune response that occurs during helminth infections is characterized by the production of large amounts of specific and non-specific IgEs [1]. However, their role is not fully understood [2]. IgEs are also induced by other stimuli, such as venom, viruses (ie influenza, herpes simplex, respiratory sincytial virus), and toxins [3]. On the other hand, IgE mediates allergic reactions, which are considered a misdirected anti-parasite response in hypersensitive people [4]. Despite major advances in our knowledge of the allergic sensitization process (i.e., molecular allergens, allergen epitope-based IgE measurements, and functional tests, such as basophil activation tests), the interpretation of an IgE response depends largely on the clinical context. The presence of IgE is not itself a predictive marker of allergies and, among patients with comparable specific IgE, some will present clinical symptoms and others will not [5]. If allergic diseases and helminth infections share some common characteristics, such as the T2 immune response, the relationships are complex. Chronic helminth infections confer protection against allergic diseases and epidemiological data have shown that their control is associated with an increased risk of developing allergic sensitization [6]. IgE induced during helminth infections is not associated with allergic symptoms, and little is known about factors that modulate the ability of IgE to trigger MC degranulation during helminth infections [7].

We evaluated the IgE and IgG4 response against environmental allergens (Dpt, Der p 1 and Der p 2) occurring during helminth infection, before and after treatment, and compared it to healthy controls. The hypersensitivity function of specific IgE was evaluated *in vitro*.

## Materials and methods

### Study population

A total of 28 subjects (aged 18–65 years) from a rural area in the periphery of Ho Chi Minh City (Vietnam) were included in this study. Eighteen of the subjects were infected with hookworm (*Necator americanus* or *Ankylostoma*. *duodenale*) and/or *Toxocara canis* infection (H+ group), and 10 were healthy controls (H- group). Diagnosis of hookworm infection was made by Kato-Katz thick smear analysis and *T*. *canis* serology (IgG detection kit from Scimedx Corporation; sample dilution 1/64). No subjects had overt symptoms of toxocariasis, and all were negative for *Ascaris lumbricoides* (Ouchterlony immunodiffusion test with home-made Ascaris (Asc) extract, serum samples concentrated 3×) and *Shistosoma stercoralis* (IgG detection kit, Scimedx Corporation; sample dilution 1/64, direct examination of feces). Exclusion criteria were pregnancy, positive skin prick test (SPT) to common aeroallergens, history of allergic and autoimmune diseases or human immunodeficiency virus infection, and previous anti-helminthic treatment during the past 6 months. Subjects from the H+ group that were infected with hookworm were evaluated at an additional visit 12 months after treatment of the infection (n = 11).

Sera from a well characterized house dust mite (HDM) allergic population (n = 9) were used as a positive control for the indirect MC activation test (MAT). This European population (aged 21–37 years) was characterized by a confirmed HDM induced asthma and positive Dpt IgE (median 58.7 (IQR 15.7–119.3) kU/L; ThermoFisher, Massachusetts, USA) [8].

### Ethics approval

The study was approved by the Ethics Committees (EC) of Pham Ngoc Thach University (Ho Chi Minh City, Vietnam, IRB-VN01013) and CHU Brugmann (Brussels, Belgium). The study was conducted in accordance with all applicable regulatory requirements and Good Clinical Practice. The ethics statement was amended by the Brugmann EC (Number 2021/72). Each participant provided signed informed consent prior to enrollment in the study. The study was registered at clinical.trials.gov (NCT02262403).

### Measurement of allergen sensitization

We compared different markers of allergen sensitization between the H+ and H- groups: IgE and IgG4 against *Dermatophagoïdes pteronyssinus* (Dpt) and its major allergens (Der p 1, Der p 2) and levels of IgE and IgG4 against Asc as a marker of immunization against nematodes. The ability of specific IgE to induce degranulation of MCs (indirect MC activation test; MAT) has been tested in the presence of the corresponding allergen (Dpt and Der p 1) in order to assess the functionality of IgE.

Blood samples (8 mL in dry tube for serum collection) were stored at -80° until assayed. Total IgE (tIgE), specific IgE, and IgG4 were measured using ImmunoCAP (Thermo Fisher Scientific, Uppsala, Sweden) according to the manufacturer’s instructions. IgE and IgG4 were tested against Dpt, Derp 1, Derp 2, and Asc as a representative of nematodes because an ImmunoCAP for hookworm or *T*. *canis* was not available [9]. A value ≥ 0.10 kU/L was considered positive for IgE and > 0.7 μg/L was considered positive for IgG4.

We also performed an ImmunoCAP ISAC 112 microarray test (Thermo Fisher Scientific, Uppsala, Sweden) that includes 112 allergen components from 51 sources. The results were analyzed on a semi-quantitative basis and expressed as ISAC standardized units (ISU) (range 0.3–100 ISU-E).

### Immunodepletion

To evaluate the interference of the glycosylated epitopes (carbohydrate cross-reactive determinant, CCD) in the detection of IgE to allergens, we repeated the measurement of Dpt and Asc using ImmunoCAP, as well as component IgE using ISAC after serum depletion of Phl p 4 antibodies, in samples exhibiting at least one positive IgE. We chose the Phl p 4 allergen to deplete anti-CCD IgE because a previous study showed that glycoproteins from pollen, such as Phl p 4, a highly glycosylated protein, were recognized by antiCCD IgE [10]. Phl p 4 ImmunoCAP were pre-washed with ImmunoCAP washing solution. We added 40 μL of serum sample from each patient to pre-washed ImmunoCAP and incubated for 30 min at room temperature. The ImmunoCAP were then centrifuged at 1700*g* for 2 min and the depleted serum was collected, pooled and immediately tested [11, 12].

### Mast cell culture

MCs were cultured as described previously by Cop et al. [13, 14]. Buffy coat cell concentrates were supplied by the Red Cross Donor Center, Belgium. Peripheral blood mononuclear cells were isolated and collected by gradient density centrifugation on Lymphoprep®. The samples were enriched for CD34+ cells using the magnetic activated cell sorting (MACS) CD34 Progenitor Isolation Kit (Miltenyi Biotec, Bergisch Gladbach, Germany) according to the manufacturer’s instructions. Enriched CD34+ cells were cultured in a serum-free methylcellulose-based medium (MethoCult SF H4236, Stemcell Technologies) supplemented with penicillin (100 U/mL; Life Technologies, Waltham, USA), streptomycin (100 μg/mL; Life Technologies), low-density lipoprotein (LDL, 10 μg/mL; Stemcell Technologies), stem cell factor (SCF, 100 ng/mL; Miltenyi Biotec, Bergisch Gladbach, Germany), and IL-3 (100 ng/mL; PeproTech, Rocky Hill, USA). Cells were plated in six-well plates at a density of 10^5^ cells/mL and cultured for 21 days at 37°C, humidified 5% CO_2_ atmosphere. We add 300 μL Iscove’s Modified Dulbecco’s Medium (IMDM; Life Technologies) containing penicillin (100 U/mL), streptomycin (100 μg/mL), 1% insulin-transferrin-selenium (Life Technologies), 0.1% bovine serum albumin (Sigma–Aldrich), SCF (20 ng/mL), and IL-3 (20 ng/mL) twice a week for 2 weeks and with IMDM containing only SCF (IMDM + SCF 10 ng/mL) the last 7 days [15].

### Immunophenotyping

On day 21 of culture, cells were retrieved, stained for viability (7-AAD) and surface makers with monoclonal anti-human CD45-BV510, CD203c-allophycocyanin (APC), CD117-fluorescein isothiocyanate (FITC), and FcɛRI-phycoerythrin (PE) to characterize the cultured MCs (BD Biosciences). Cultured mature human MCs were defined as CD45+, CD117+, and CD203c+low cells. Flow cytometric analysis was performed on a FACSCanto II flow cytometer (BD Immunocytometry Systems, San Jose, CA) and data analyzed using BD FACSDiva^TM^ software (BD Biosciences, San Jose, CA, USA).

### Indirect mast cell activation assay

The function of IgE was evaluated by passively sensitizing cultured MCs with allogenic serum from H-, H+, and HDM-allergic subjects [14]. Cells were incubated with undiluted serum (30 min, 37°C, humidified 5% CO_2_ atmosphere), centrifuged (200*g*, 5 min, 20°C), and the cell pellet resuspended in Tyrode’s buffer (Sigma-Aldrich) at a concentration of 0.5 × 10^6^ cells/mL. A 100 μL of the MC suspension was activated with 100 μL Tyrode’s buffer as a negative control, 100 μL of 2 μg/mL monoclonal anti-IgE (BD Biosciences) as a positive control, 100 μL of Dpt/*D*. *farinae* (Df) extract (5000 AU/mL (10^−3^) Glycotope, BD Biosciences), or 100 μL of 2.5 μg/mL Der p 1 (Indoor Biotechnologies Ltd, LTN-DP1-1) for 20 min at 37°C. Reactions were stopped on ice and cells stained with 7-AAD, monoclonal antihuman CD45-BV510, CD203c-APC, CD117-BV421, and CD63-PE-Cy7. The expression of CD63 (% of viable CD117+CD203c+low) was measured as an indicator of MC activation. The AR was calculated as the expression of CD63 after stimulation with anti-IgE, Dpt, or Der p 1, divided by the expression of CD63 in the unstimulated condition [16, 17]. To increase the level of serum IgE used for passive sensitization, sera were concentrated 3.2 ± 0.2-times using an Amicon ultra-filtration tube and centrifuging at 4000*g* for 15 min. Passive sensitization followed by activation was repeated.

### Statistical analysis

Statistical analyses were performed using GraphPad Prism software (version 6.0; Graph Pad Software). Data were expressed as the median with interquartile range (IQR) and analyzed by a non-parametric Mann-Whitney test to compare unpaired data from two groups. Alternatively, the Wilcoxon test was used for paired data and Kruskal-Wallis test with corrected Dunn’s test for multiple comparisons. Correlations between antibody responses were calculated using the Spearman correlation. P ≤ 0.05 was considered significant.

## Results

### Dpt and Asc humoral response

**Fig 1:**
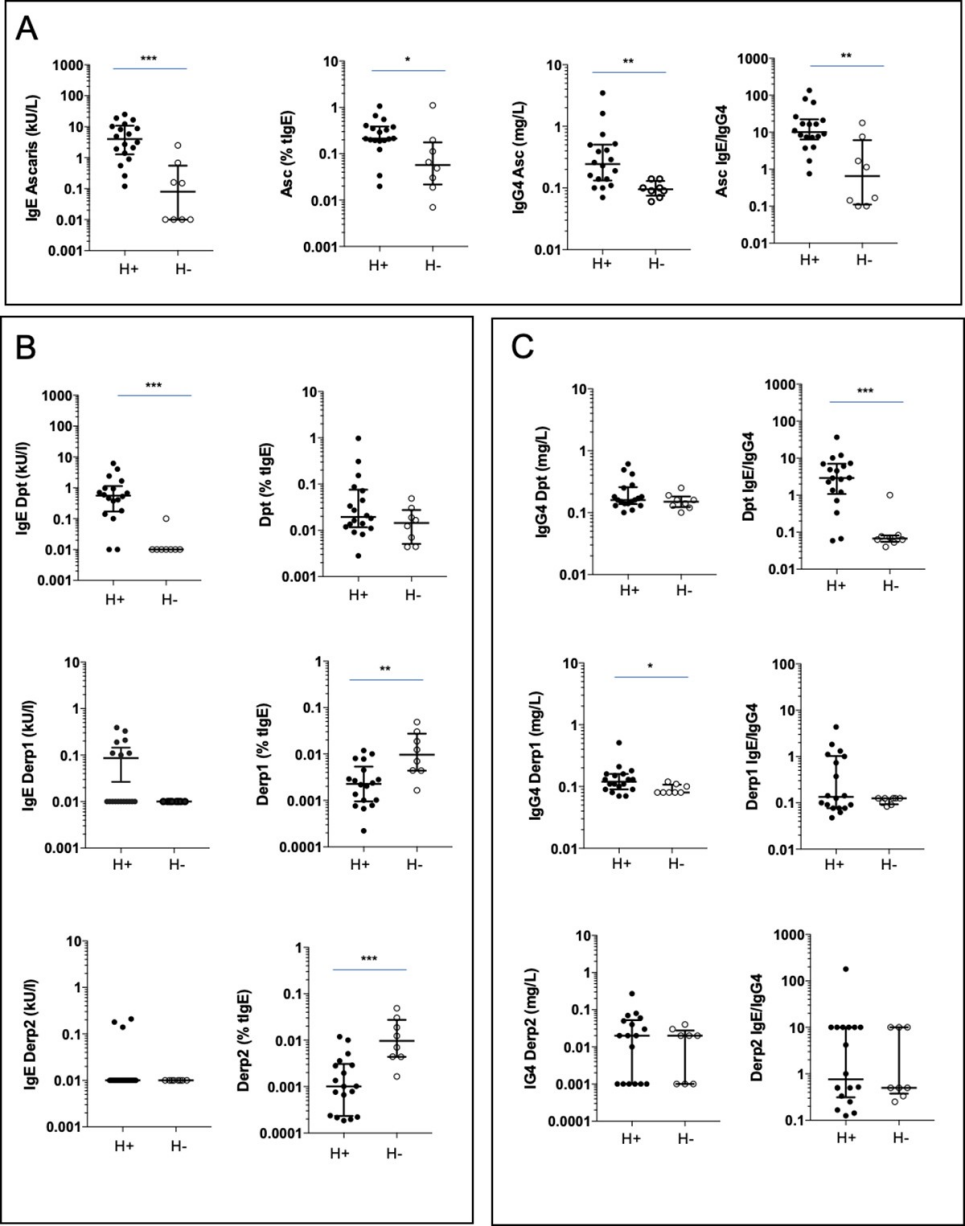
House dust mite and Ascaris IgE and IgG4 responses. IgE and IgG4 against Ascaris (A), IgE (B), and IgG4 (C) against *D*. *pteronyssinus* (Dpt), Derp1, and Derp2 in subjects with (H+) and without (H-) helminth infection. *p<0.05, **p<0.01, ***p<0.001, Mann-Whitney test.

As expected, helminth infection was associated with a higher tIgE level (median 1409 (IQR 640–4745) vs. 112.1 (IQR 38.1–227.5) kU/L, p<0.001) in the H- group. The Asc response was characterized by higher Asc IgE, Asc IgE/tIgE ratio, Asc IgG4, and Asc IgE/Asc IgG4 ratio (Fig 1A).

Dpt IgE was also significantly increased in the H+ group, whereas IgEs against components, such as Der p 1 and Der p2, were not significantly different (Fig 1B). The specific Dpt IgE/tIgE ratio was not different among H+ and H- subjects, and was lower for Der p 1 and Der p 2 components in the H+ group. Der p1 IgG4 was significantly higher in the H+ group, as well as the Dpt IgE/Dpt IgG4 ratio (Fig 1C). The Asc IgE and IgG4 levels positively correlated with tIgE and Dpt, Der p 1, and Der p 2 IgE (S1 Fig).

Eleven subjects infected with hookworm were re-evaluated 1 year after treatment. Two (18%) developed positive SPT against HDM (Dpt, Df, and *B*. *tropicalis* in one subject, Df and *B*. *tropicalis* in the other subject). Compared to non-sensitized subjects, both subjects seemed to exhibit higher Dpt IgE, Dpt IgE/tIgE, and Dpt IgE/Dpt IgG4 ratio (S2 Fig). Their Asc response was also different, with higher Asc IgE.

### Component sensitization profile

**Fig 2:**
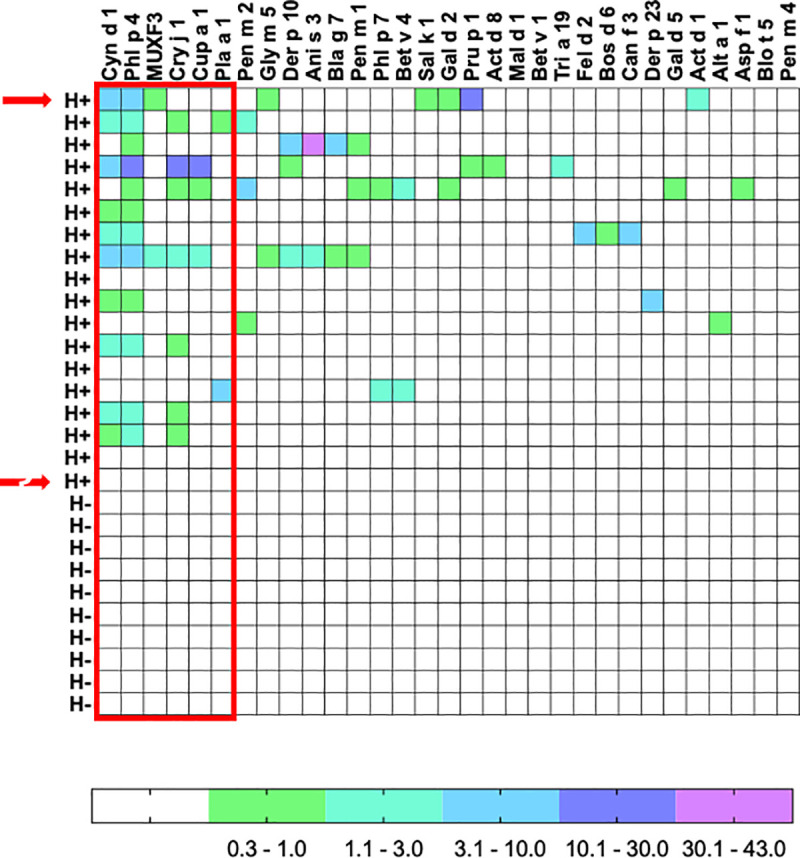
Specific IgE repertoire determined by ISAC. Specific IgE for components measured by the ISAC microarray. Subjects with (H+) and without (H-) helminth infection and allergens with at least one positivity above 0.3 ISU are depicted. Positive results are expressed in ISAC standardized units (ISU) and categorized according to arbitrary ranges. The red framework shows the glycosylated allergens. Red arrows indicate both subjects that developed positive SPT 12 months after treatment.

The specific IgE repertoire of each subject determined by ISAC microarray measurements is shown in Fig 2. Fourteen of the 18 (78%) H+ subjects recognized at least one component, compared to none in the H- group (chi-squared test p<0.0001). Among Dpt components available on ISAC, we observed positive results for Der p 10 (n = 3) and Der p 23 (n = 1), but none for Der p1 or Der p2. Seventy different components were recognized at different levels of intensity (0.30 to 42.53 ISU). The most often recognized antigens (36/70) were Phl p 4 > Cyn d 1 > Cry j 1 > Cup a 1 > MUXF3 and Pla a 1. All these allergens have the common caractéristics of bearing CCD, such as β-1,2-xylose and/or ⍺-1,3-fucose substituted N-glycans, unlike other natural allergens present on the microchip [10]. Other components belong to different animal and vegetal protein families that exhibited different homologies with parasites antigens (arginine kinase, tropomyosin, beta-conglycin, polcalcin, pectin methylesterase, PR-10, serum albumin, ovalbumin, among others). No α-Gal sensitization was observed, and this was confirmed with ImmunoCAP.

### Mast cell characterization

Twenty-one days after plating, all cultured were recovered and analysed by flowcytometry. CD45-positive gated cells had 75 ± 10.5% of viability. As demonstrated by Cop et al. [13], two clear distinct cell populations were identifiable: CD117+CD203c+low cells and CD117‐CD203c+low cells. Almost 80% of the CD117+CD203c+low cells expressed FcɛRI. A representative sample for cell immunophenotyping is illustrated in S3 Fig. Cultured mature human MCs were defined as CD45+, CD117+, CD203c+low cells.

### IgE-mediated activation

**Fig 3:**
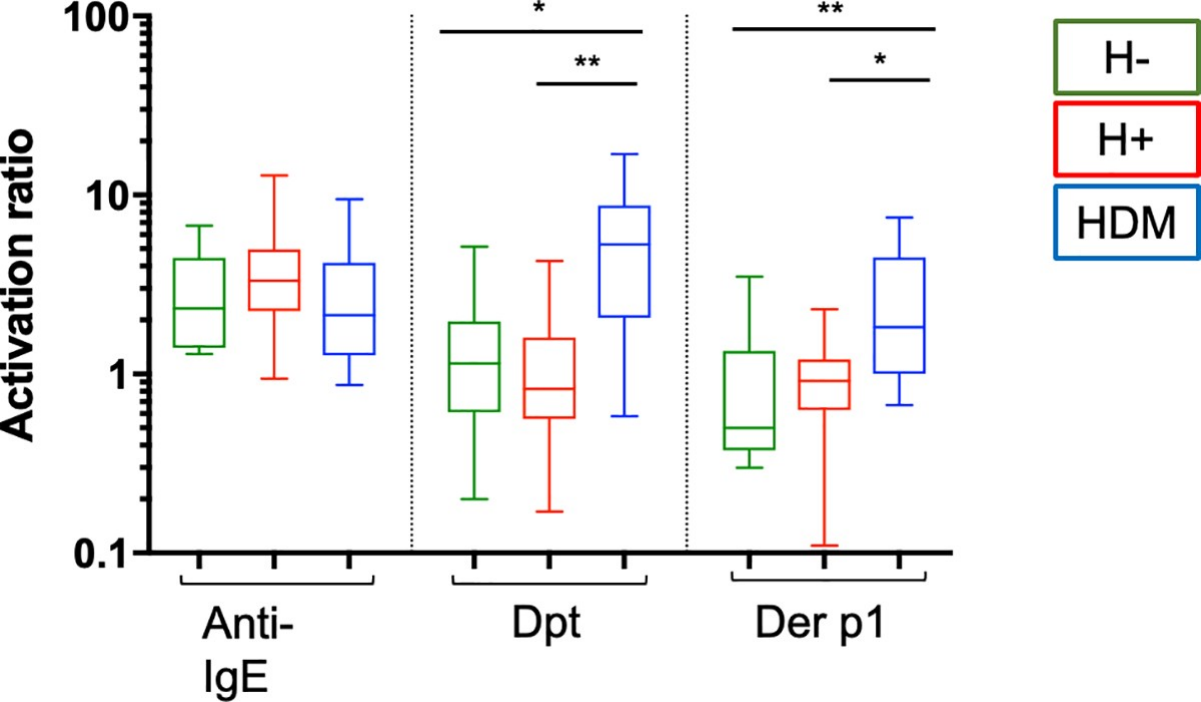
Mast cell activation after passive sensitization. The activation ratio (AR) calculated between CD63 (%) after anti-IgE, Dpt (*Dermatophagoides* extract), Der p1 stimulation, and negative control. H-: non-infected group (green, n = 11), H+: helminth-infected group (red, n = 18), HDM: subjects allergic to house dust mites (blue, n = 12). *p <0.05, **p < 0.01, ***p < 0.001, Kruskal-Wallis test with corrected Dunn’s test.

The ARs are shown in Fig 3. The specific stimulation with Dpt/Df extract and Der p1 allergen induced a significantly higher AR in the HDM allergic population compared to the H+ and H- groups, whereas the H+ group did not exhibit any difference compared to the H- group. Passive sensitization was repeated with concentrated sera to evaluate the role of an increased IgE level and to sensitize the test. However, no change was observed on MC activation (S4 Fig).

### Interference of CCDs in IgE sensitization

**Fig 4:**
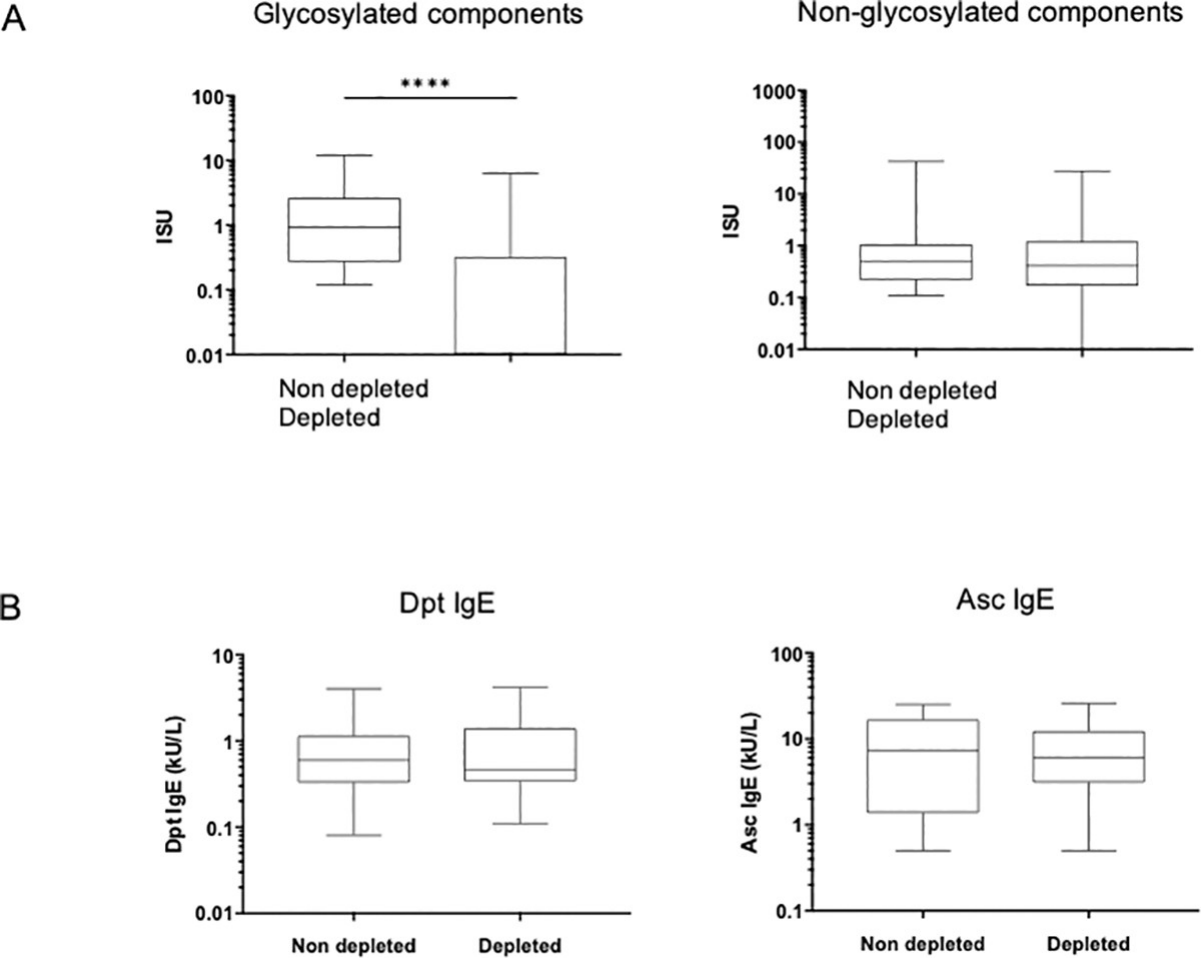
Effect of Phlp4 IgE depletion on the levels of IgE. A. Specific IgE against components before (Non-depleted) and after depletion (Depleted) with Phl p4 as measured by ISAC for glycosylated (Phl p 4, Cyn d 1, Cry j 1, Cup a 1 > MUXF3 and Pla a 1) allergens and non-glycosylated allergens (107 other components present on the ISAC microchip). B. Specific IgE against *D*. *pteronyssinus* (Dpt) and Ascaris (Asc) using ImmunoCAP before and after depletion with Phl p 4. ***p<0,001, before vs. after depletion, paired Wilcoxon test.

Phl p4 depleted sera recognized only 13 glycosylated components as compared with 36 with undepleted sera (S5 Fig). The levels of IgE against glycosylated allergens were significantly reduced (p<0.0001), whereas the levels of IgE directed against non-glycosylated allergen remained unchanged (Fig 4A). Dpt and Asc IgE levels were not affected by Phl p 4 IgE depletion (Fig 4B).

## Discussion

One of the unanswered questions in allergology is what determine the ability of IgE to trigger clinical symptoms.

### Helminth response

Our study confirmed that the H+ group exhibited the characteristics of a type T2 response against helminths, i.e., a high level of tIgE and parasite-specific IgE and IgG4 [18]. We consider the IgE Asc a marker of sensitization to helminths resulting from a cross reaction between helminth antigens (i.e., hookworm and/or *T*. *canis*) because *A*. *lumbricoides* infection was excluded by feces and serological negative results.

### HDM sensitization

First, we investigated the response against Dpt as representative of HDMs that are a major cause of allergic rhinitis and asthma [19] and the most often sensitizer after deworming in soil-transmitted helminthiasis-endemic region, such as Vietnam [6]. H+ subjects exhibited IgE against Dpt global allergen extract. Major and minor components, such as Der p 1, Der p 2, Der p 10, and Der p 23, were elevated in some H+ subjects, but the difference from the H- group was not significant (ImmunoCAP and ISAC). Consequently, we were not able to identify components explaining the Dpt sensitization. Up to 30 allergens from Dpt have been described, and it is possible that the Dpt IgE present in our H+ patients target other components (glycosylated or proteic). Moreover, allergen extract do not contain all the allergen components.

The discrepancies between results from ISAC and ImmunoCAP may be explained by technical differences (see S1 Table for raw data). Compared to ISAC, ImmunoCAP is more sensitive and less suscetible to interference other antibodies, such as IgG [20], but may be influenced by IgE anti-CCD binding to carbohydrate-based solid phases [21].

The positive correlation between total allergens and Asc IgE levels suggests a non-specific polyclonal stimulation effect as the origin of the IgE production (a class effect) rather than a specific allergen sensitization induced by helminth infection.

### Sensitization to CCDs

The most often glycosylated antigen recognized was Phl p 4, which was quite surprising as MuxF3 the genuine marker of CCD sensitization was negative for most of them. However, Phl p 4 can be glycosylated at four different sites and, is therefore, a highly cross-reactive CCD-bearing allergen [22]. After depletion of IgE against Phl p 4, the IgE levels against glycosylated antigens decreased significantly, meaning that these positive results were due, at least in part, to the CCD. It confirmed that IgE produced during helminth infection mainly recognizes CCD+ allergens especially β-1,2-xylose and/or ⍺-1,3-fucose substituted N-glycans.

The IgE Dpt and Asc level were not affected by the Phl p 4 depletion, confirming results from Hamid et al. who showed that IgE targeting core β-1,2-xylose and/or ⍺-1,3-fucose substituted N-glycans does not play a role in Dpt and Asc sensitization [23]. However, N-glycans are not unique CCDs, and other carbohydrate species may exist in Dpt extract [24].

Glycans are produced by helminths during infection, among a large panel of molecules that can induce and regulate the T2 response [25] and their role remains largely unknown.

There is broad agreement from experimental studies and clinical practice that IgEs against CCDs lack clinical relevance [26]. From a hygiene hypothesis perspective, IgE against CCD epitope are thought to play a role in the protective effect of helminth infection against allergy. Recently, reactivity to a subset of N-glycans was shown to protect from asthma [27].

Another interesting finding is the absence of sensitization to carbohydrate epitope galactose-α 1,3-galactose (α-Gal). This contradicts the study by Arkestål et al. [28] suggesting that schistosomiasis and/or soil-transmitted helminthiasis (*A*. *lumbricoides*, *T*. *trichiura*, or hookworm infection) in Zimbabwe induced sensitization to α-Gal. This contradictory aspect could be explained the different helminthiasis affecting both population, or by a confounding factor inducing α-Gal sensitization not related to helminth infection (ie ectoparasites). *T*. *canis* may inhibit production of IgE to α-GAL [29] but in our study, very few subjects were infected with *T*. *canis*.

### Proteic components

Other components recognized by IgE in H+ subjects belong to several usual protein allergen families, such as PR10, arginine kinase, tropomyosin, serum albumin, and polcalcin, suggesting that helminth infection may induce sensitization to proteins that are well known allergens from clinically relevant sources. However, none of the subjects presented any cutaneous sensitization against common respiratory allergens or clinical symptoms of allergy. In contrast to El-Faham et al.’s report [24], our analysis of molecular IgE sensitization allows us to suggest that CCD cross-reactivity is not the unique mechanism of allergen sensitization during helminth infection, and that sensitization against peptidic epitopes occurs.

### Function of Dpt IgE

We investigated the reactivity to Dpt allergens using an *in vitro* model of MC passive sensitization. Overall, MC incubated with Dpt IgE positive sera from H+ subjects failed to degranulate in presence of Dpt.

Either, Dpt IgE present in H+ individuals may not be functional, or are only weakly functional. Another explanation could be the low level of anti-Dpt specific IgE suggesting that the occupation of FcεRI on MC was too low to be efficiently cross-linked. We attempted to increase the level of IgE by concentrating sera, but the AR was not different.

The recognition of CCDs in the extract could explain the absence of activation. Indeed, sensitization to CCDs has been described during helminth infection [23, 27, 30–33] and, with the exception of α-Gal [34], CCD-specific IgE are unable to trigger MCs or basophil degranulation *in vivo*. However, *in vitro* binding to anti-CCD IgE can result in cross-linking of FcεRI and activation of MCs, but it requires much higher concentrations [35].

An alternative hypothesis is that IgE from H+ subjects are not or less able to initiate activation compared to allergic subjects because of a different glycosylation pattern of the antibody itself. Indeed, the sialylation of IgE was recently described to be a regulator of allergic disease [36]. To the best of our knowledge, the glycosylation of IgE during helminth infection is still unknown.

Finally, serum from H+ subjects could contains inhibiting factors. Antigen-specific IgG4 is linked to the modified T2 response, and the allergen-driven T2 response characterized by a high IgG4/IgE ratio has been proposed to result in immunological tolerance [37, 38]. In our study, the levels of Dpt and Der p 2 IgG4 were not significantly higher during helminth infection. Only Der p 1 IgG4 was higher, and it may be the result of homologous structures between the cysteine protease of Dpt and helminths [39]. Moreover, we did not observe any correlation between IgG4 and AR with the corresponding stimulus (data not shown). This suggests that IgG4 is not a major factor of protection for MC degranulation against Dpt in our study. The role of other isotypes (i.e., IgG1) or inhibitory factors cannot be excluded.

### Evolution of the humoral response after treatment of infection

When looking at the subgroup of subjects infected with hookworm evaluated 12 months after treatment, two developed a positive SPT against HDMs. Despite the limited number of subjects and the absence of a control group, it is of interest to note that both subjects presented differences compared to subjects who did not develop a positive SPT. At baseline (SPT-negative), these two subjects already had a stronger response against Dpt IgE and Asc that persisted after treatment of the infection. It suggests that subjects that become HDM-sensitized already had a stronger T2 response against both Asc and Dpt. The absence of cutaneous sensitization against HDM may be due to the tolerogenic environment of the “modified” T2 response, such as regulatory T cells [40, Personal communication], IL10, and alternatively activated macrophages [7] induced during helminths, that disappears when the infection is cured. In addition, the Dpt IgE/tIgE ratio seemed to increase in these patients after hookworm treatment. This is in accordance with data observed in allergic diseases, showing that a high allergen-specific IgE/tIgE ratio can predict allergy in sensitized children [41, 42]. Taken together, the findings suggest that the intensity of sensitization to HDM is linked to the intensity of the T2 response to helminths and, perhaps, to a predisposition for developing a strong T2 response.

Overall, subject to the small number of subjects, our data indicate that the IgE response occurring during helminth infection is characterized by the production of non-functional IgE directed against environmental allergens, such as HDMs, N-glycans (CCDs), and peptidic allergens from different families. Sensitization to Dpt was not explained by sensitization to N-glycans nor to major allergens, was not able to induce MC activation, and was strongly linked to the intensity of the T2 response against helminths.

## Supporting information

S1 Fig Humoral response correlation matrix. Correlation between IgE and IgG4 response against Ascaris (Asc), D. pteronyssinus (Dpt), Der p1 and Der p2 were calculated with the Spearman correlation test for all the samples (n = 28) at T0. The Spearman r value is depicted in the figure and the correspondent p value is represented by the green scale when ≤ 0.05 and in white when non-significant.
(TIF)

S2 Fig Dpt and Ascaris response before and after treatment of infection. T0: time of inclusion, 12M: 12 months after the hookworm treatment. Among H+ subjects that were evaluated at T0 and 12M (n = 11), wo groups were defined based on results from SPT 12M after the treatment of infection: SPT+ subjects are represented with squares and SPT–subjects with circles.
(TIF)

S3 Fig Representative sample of flow cytometry phenotyping. A: Mast cells gating strategy. Mast cells are defined as CD45+ alive and CD117+CD203c+low cells. Expression of FcɛRI is shown on the histogram in blue for CD117+CD203c+low cells and green for CD117‐CD203c+low. B: The activation of mast cells was measured by expression on CD63 on CD117+CD203c+low cells. The positive threshold was placed on the unstimulated condition.
(TIF)

S4 Fig Effect of serum concentration on MAT. Activation ratio calculated between CD63 (%) after Anti-IgE, Dpt, Der p 1 stimulation and negative control. H-: non infected group (n = 3), H+: helminth infected group (n = 9) and House dust mite (HDM) group: subjects allergic to mites (n = 5). P: pur serum and C: concentrated serum. Comparison between P and C were tested with a Wilcoxon test. A p value ≤ 0.05 was considered significant (*p < 0.05, **p < 0.01, ***p < 0.001).
(TIF)

S5 Fig Effect of Phl p 4 IgE depletion on components IgE sensitization measured with ISAC IgE sensitization profile measured with microarray ISAC in subjects with (H+) helminths infection after ImmunoCAP depletion with Phl p 4. Results are expressed in ISAC Standardized Units (ISU), considered positive when above 0.30 ISU and categorized according to arbitrary ranges. Only subjects with at least one positivity were tested after Phl p 4 depletion. Red framework shows the glycosylated allergens according to manufacturer.
(TIF)

S1 Table IgE against Der p 1 and Der p2 with ImmunoCAP and ISAC. (PDF)

## Abbreviations

α-GAL: galactose alpha 1,3 galactose
CCD: carbohydrate cross-reactive determinant
Df: *Dermatophagoïdes farinae*
Dpt: *Dermatophagoïdes pteronyssinus*
FcεRI: IgE high affinity receptor
HDM: house dust mite
IgE: immunoglobulin E
IgG4: immunoglobulin G4
IL: interleukin
ISU: ISAC standardized units
H+: helminth infected group
MC: mast cell
SPT: skin prick test
