# Supplementary figures and images for: Helminth infection induces non-functional sensitization to house dust mites
Source: PLoS One. 2021 Jul 1;16(7):e0253887. doi: 10.1371/journal.pone.0253887 (PMC8248592; doi:10.1371/journal.pone.0253887)

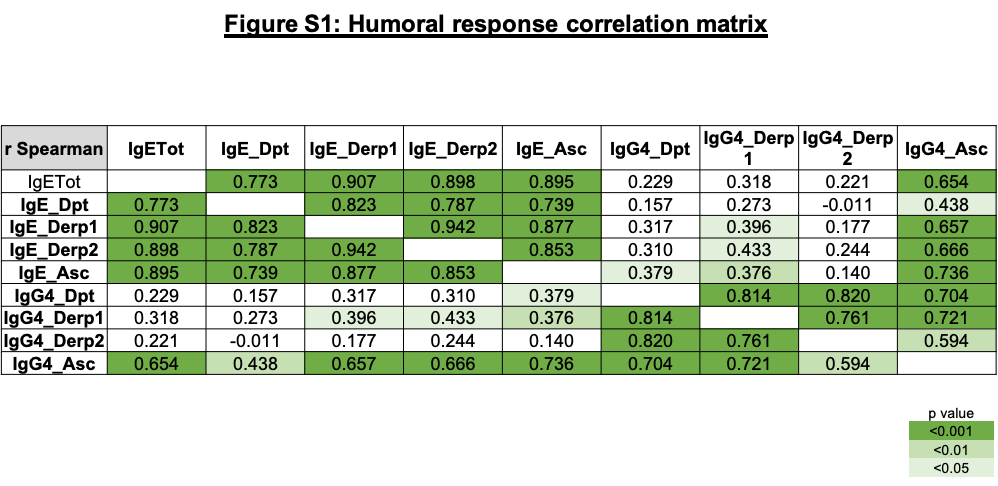

Supplement: S1 Fig — Correlation between IgE and IgG4 response against Ascaris (Asc), D. pteronyssinus (Dpt), Der p1 and Der p2 were calculated with the Spearman correlation test for all the samples (n = 28) at T0. The Spearman r value is depicted in the figure and the correspondent p value is represented by the green scale when ≤ 0.05 and in white when non-significant. (TIF) [file pone.0253887.s001.tif]

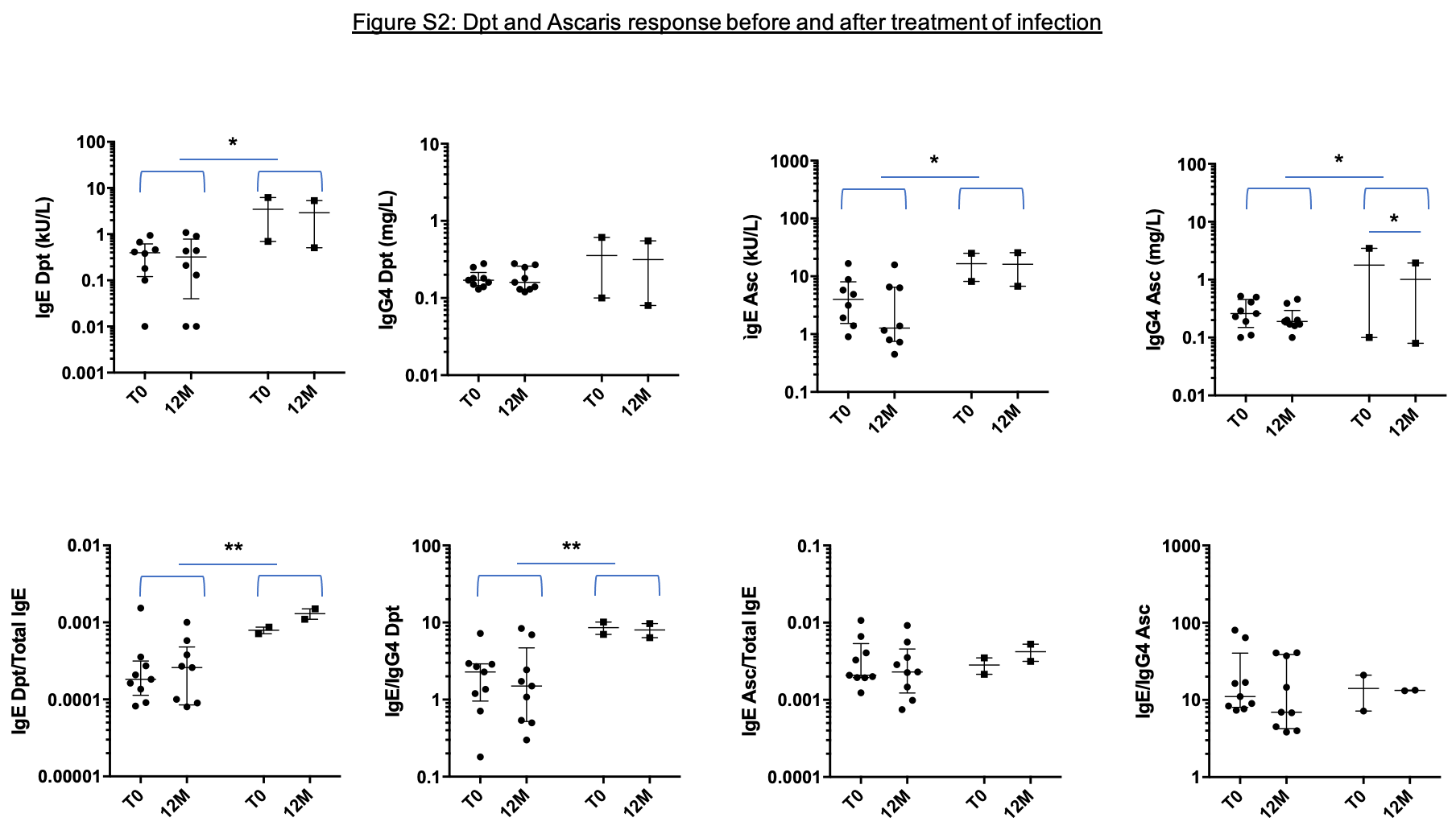

Supplement: S2 Fig — T0: time of inclusion, 12M: 12 months after the hookworm treatment. Among H+ subjects that were evaluated at T0 and 12M (n = 11), wo groups were defined based on results from SPT 12M after the treatment of infection: SPT+ subjects are represented with squares and SPT–subjects with circles. (TIF) [file pone.0253887.s002.tif]

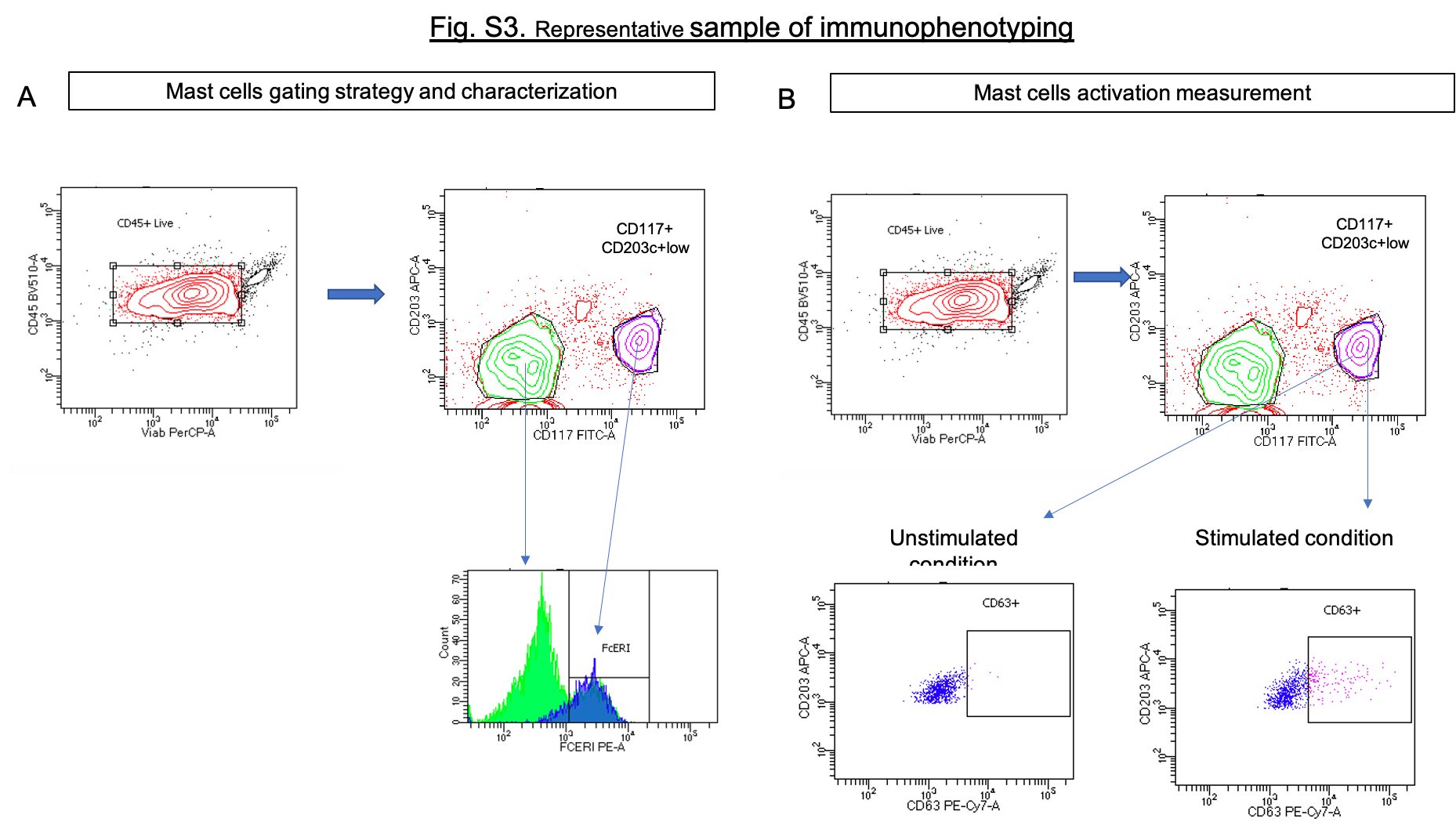

Supplement: S3 Fig — A: Mast cells gating strategy. Mast cells are defined as CD45+ alive and CD117+CD203c+low cells. Expression of FcɛRI is shown on the histogram in blue for CD117+CD203c+low cells and green for CD117‐CD203c+low. B: The activation of mast cells was measured by expression on CD63 on CD117+CD203c+low cells. The positive threshold was placed on the unstimulated condition. (TIF) [file pone.0253887.s003.tif]

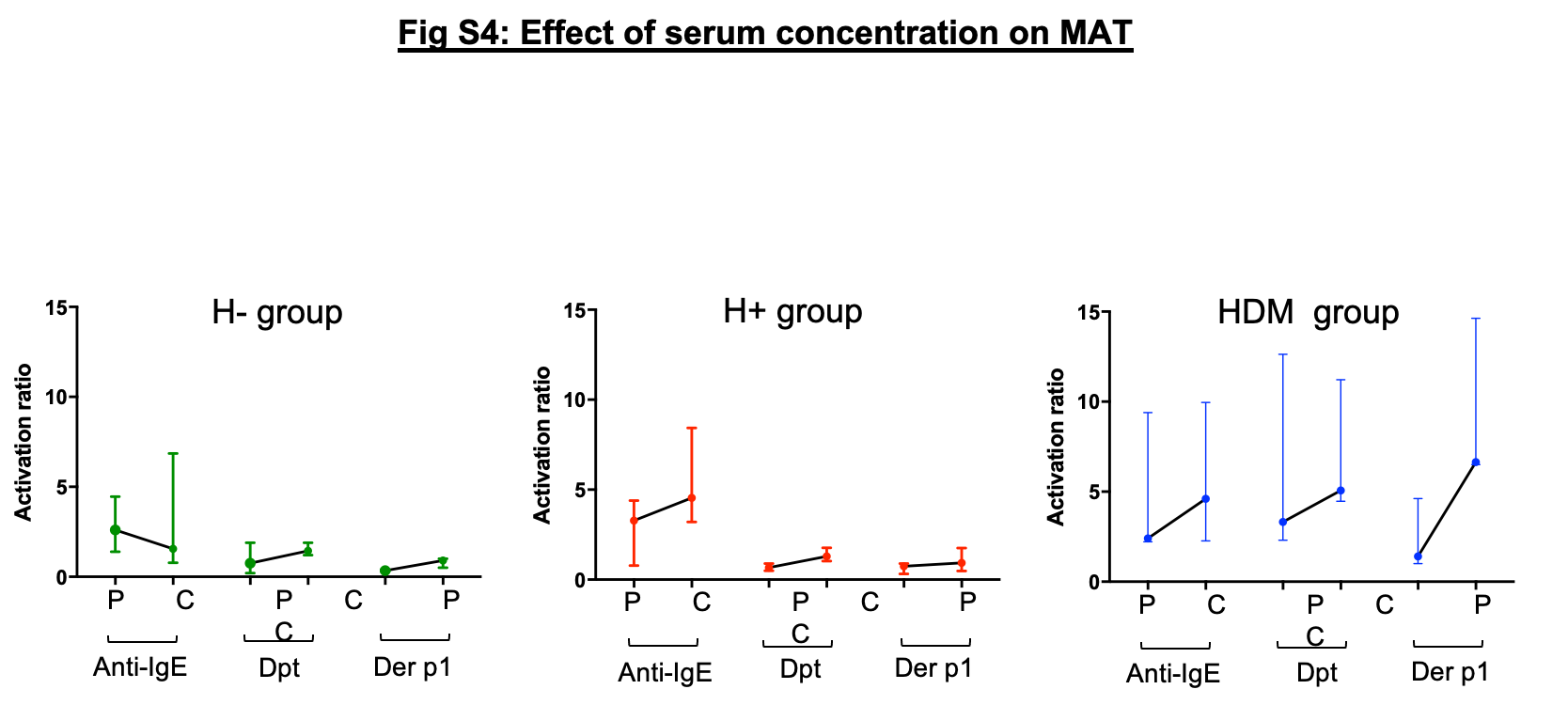

Supplement: S4 Fig — Activation ratio calculated between CD63 (%) after Anti-IgE, Dpt, Der p 1 stimulation and negative control. H-: non infected group (n = 3), H+: helminth infected group (n = 9) and House dust mite (HDM) group: subjects allergic to mites (n = 5). P: pur serum and C: concentrated serum. Comparison between P and C were tested with a Wilcoxon test. A p value ≤ 0.05 was considered significant (*p < 0.05, **p < 0.01, ***p < 0.001). (TIF) [file pone.0253887.s004.tif]

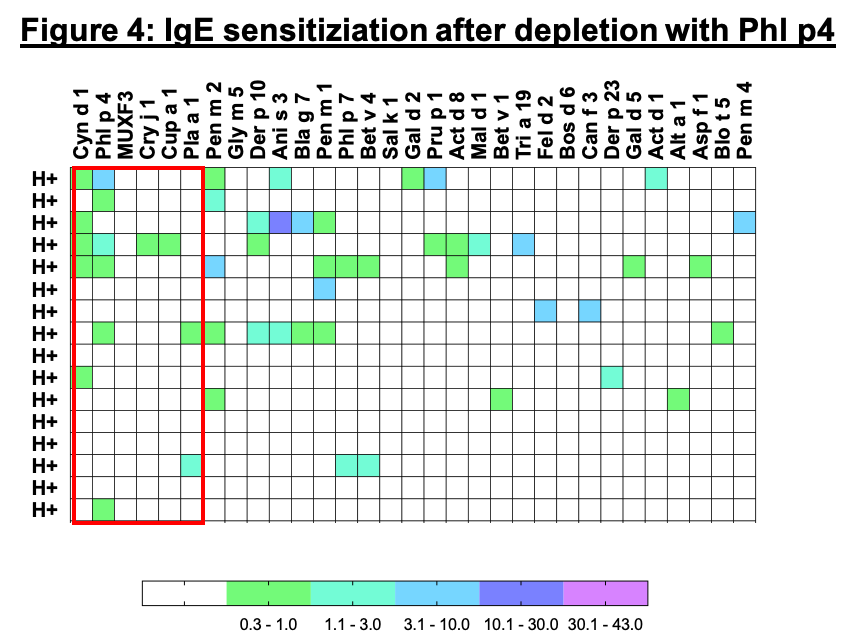

Supplement: S5 Fig — Results are expressed in ISAC Standardized Units (ISU), considered positive when above 0.30 ISU and categorized according to arbitrary ranges. Only subjects with at least one positivity were tested after Phl p 4 depletion. Red framework shows the glycosylated allergens according to manufacturer. (TIF) [file pone.0253887.s005.tif]
