# Supplementary material for: Helminth infection induces non-functional sensitization to house dust mites
Source: PLoS One. 2021 Jul 1;16(7):e0253887. doi: 10.1371/journal.pone.0253887 (PMC8248592; doi:10.1371/journal.pone.0253887)
Supplement: S1 Table — (PDF) [file pone.0253887.s006.pdf]

S1 Table: IgE against Der p 1 and Der p2 with ImmunoCAP and ISAC

H Infection	Derp1 (kU/L)	Derp1 (ISU)	Derp2 (kU/L)	Derp2 (ISU)
H+	0.39	0.00	0.18	0.00
H+	0.01	0.00	0.00	0.00
H+	0.21	0.00	0.06	0.00
H+	0.04	0.00	0.01	0.00
H+	0.06	0.00	0.03	0.00
H+	0.33	0.00	0.21	0.00
H+	0.02	0.00	0.01	0.00
H+	0.04	0.00	0.02	0.00
H+	0.04	0.00	0.02	0.00
H+	0.19	0.00	0.14	0.00
H+	0.10	0.00	0.06	0.00
H+	0.11	0.00	0.08	0.00
H+	0.03	0.00	0.01	0.00
H+	0.03	0.00	0.02	0.00
H+	0.11	0.00	0.08	0.00
H+	0.01	0.00	0.00	0.00
H+	0.07	0.00	0.03	0.00
H+	0.07	0.00	0.06	0.00
H-	0.01	0.00	0.00	0.00
H-	0.02	0.00	0.00	0.00
H-	0.01	0.00	0.00	0.00
H-	0.03	0.00	0.02	0.00
H-	0.01	0.00	0.00	0.00
H-	0.01	0.00	0.00	0.00
H-	0.01	0.00	0.00	0.00
H-	0.02	0.00	0.00	0.00

Comparison of raw data for helminths infected (H+) and non-infected (H-) subjects measured with ImmunoCAP and with ISAC. Results are expressed in kU/L for ImmunoCAP and ISAC standardized units (ISU) for ISAC.
